# A Novel Approach: Combining Prognostic Models and Network Pharmacology to Target Breast Cancer Necroptosis-Associated Genes

**DOI:** 10.3389/fgene.2022.897538

**Published:** 2022-08-22

**Authors:** Congzhi Yan, Conghui Liu, Zhixuan Wu, Yinwei Dai, Erjie Xia, Wenjing Hu, Xuanxuan Dai

**Affiliations:** Key Laboratory of Clinical Laboratory Diagnosis and Translational Research of Zhejiang Province, Department of Breast Surgery, The First Affiliated Hospital of Wenzhou Medical University, Wenzhou, China

**Keywords:** immune microenvironment, necroptosis, network, breast cancer, treatment

## Abstract

Breast cancer (BC) accounts for the highest proportion of the all cancers among women, and necroptosis is recognized as a form of caspase-independent programmed cell death. We created prognostic signatures using univariate survival analysis, and lasso regression, to assess immune microenvironments between subgroups. We then used network pharmacology to bind our drugs to target differentially expressed genes (DEGs). A signature comprising a set of necroptosis-related genes was established to predict patient outcomes based on median risk scores. Those above and below the median were classified as high-risk group (HRG) and low-risk group (LRG), respectively. Patients at high risk had lower overall survival, and poorer predicted tumor, nodes, and metastases stages (TNM). The novel prognostic signature can effectively predict the prognosis of breast cancer patients docking of *β,β-dimethyl acryloyl shikonin (DMAS)* to possible targets to cure breast cancer. We found that all current prognostic models do not offer suitable treatment options. In additional, by docking drugs DMAS that have been initially validated in our laboratory to treat breast cancer. We hope that this novel approach could contribute to cancer research.

## Introduction

Necroptosis,a novel type of cell death regulated through mechanisms that do not depend on cystathionine, is a guarder of defense against some invasions. Necrotizing apoptosis might further fuel apoptosis and enhance anti-tumor immunity in patients (Schwabe and Luedde, 2018). In addition, we found that *PPm1b*(Frontiers Editorial Office, 2021) and *ZBP1* (Baik et al., 2021)have now been shown to induce apoptosis in breast cancer cells *via* the necroptosis pathway. Although a number of genes have been identified, the mechanism of action of necroptosis in breast cancer is complex. The complete mechanism needs to be further explored.

Breast cancer is one of the most malignant human diseases in the world. (Britt et al., 2020). The global incidence of BC increased at an annual rate of 3.1%, from 641,000 in 1980 to 1.6 million in 2010 (Flemming et al., 2021). Treatment decisions for various BC subtypes have recently been guided by the results of microarrays, high-throughput sequencing, multi-gene prediction, gene tests, and 21-gene recurrence scores (Kawaji et al., 2021). Polygenic prediction is applied worldwide to predict the effects of chemotherapy or patient prognosis (Elliott et al., 2020). However, the treatment for BC remains a seemingly insurmountable challenge because of the dearth of therapeutic targets and biomarkers. Several necroptosis-related genes (NRGs) have been identified as possible therapeutic targets for BC. Of note, *β,β-dimethyl acryloyl shikonin* is an anticancer compound extracted from *Lithospermum erythrorhizon roots* (Xuan and Hu, 2009). Several studies have highlighted the potential clinical relevance for *DMAS*. For instance, apoptosis in lung cancer cells by activating the p38 pathway, lung adenocarcinoma can avoid this *via* endoplasmic reticulum stress-mediated autophagy (Wang and Ma, 2015). The apoptosis of melanoma is induced by *DMAS* by upregulating phorbol-12-myristate-13-acetate-induced protein 1 (*NOXA*) (Stallinger et al., 2020), and *DMAS* blocks hepatocellular carcinoma cell cycle arrest in the G2 phase (Tummers et al., 2020). However, the role of *DMAS* in BC remains unclear. *DMAS*, a potential drug to treat breast cancer, was applied to treat BC in network pharmacology.

Cyberpharmacology is a useful tool to discover candidate disease targets and mechanisms of bioactive components for treating diseases. At present, many prognostic models simply predict the therapeutic effect of drugs. In this study, we investigated prognostic models to predict the prognosis of breast cancer patients. The mechanism of action of DMAS, at the same time, is described and the targets are mapped in breast cancer. Notably, necroptosis -related genes prognostic model has not yet been reported. We need to predict the prognosis of patients and also need drugs to improve the prognosis of patients. The biggest innovation of this study is the addition of DMAS docking disease targets to the prognostic model. This may hopefully improve the prognosis of breast cancer patients.

## Materials and Methods

### Data Collection and Identification of NRGs

**FIGURE 1:**
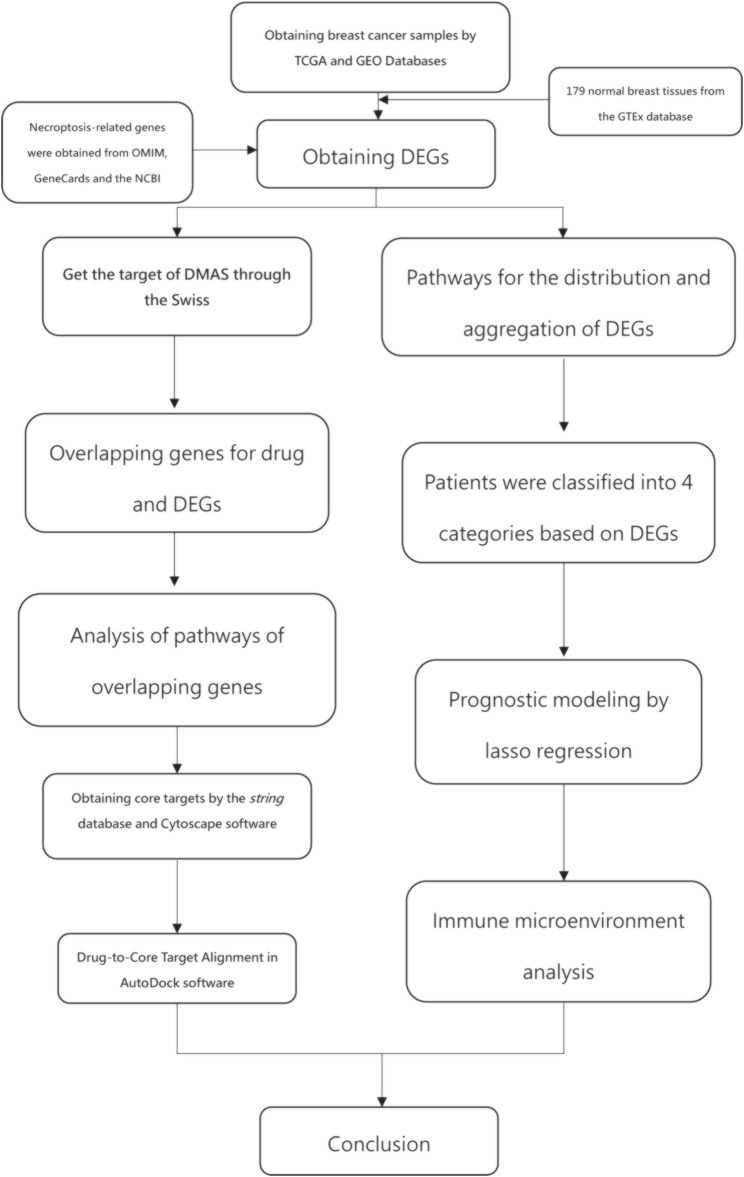
work of flow in this study.

Figure 1 shows the work of flow in this study. We first obtained the sample information from the database to derive DEGs, and based on the DEGs, we built a prognostic model. After this, core targets are screened from the DEGs and DMAS is docked to these targets. The genetic matrix associated with breast cancer was downloaded from The Cancer Genome Atlas (TCGA,https://portal.gdc.cancer.gov/) in public databases (Table 1). Genes associated with necroptosis were accessed from the GeneCards https://www.genecards.org/), OMIM databases (https://omim.org/) and the NCBI gene function module (https://www.ncbi.nlm.nih.gov/). These databases are frequently used to find pathway-related genes. The GSE20685 and GSE21653 datasets (Table 1) were obtained from the public Gene Expression Omnibus (GEO, https://www.ncbi.nlm.nih.gov/geo/). The TCGA dataset contains all types of breast cancer patients, and the GSE20685 is containing all types of breast cancer patients. GSE21653 is a gene expression signature identifies two prognostic subgroups of basal breast cancer that possesses a favorable prognosis. The local ethics committee waived approval for this study, which conformed to GEO and TCGA data access and release policies. Differentially expressed genes associated with necroptosis (NDEGs) were analyzed and DEGs were classified to distinguish different types of population survival. To further validate the differentially expressed genes, we added 179 normal breast tissues from the Genotype-Tissue Expression Project (GTEx, https://commonfund.nih.gov/GTEx/). *The “limma” package in R* was used to identify differentially expressed NDEGs by comparing gene expression between tumor and adjacent normal tissue in the TCGA cohort (log FC > 0.5, FDR <0.05).

**TABLE 1:** Data of all patients.

	TCGA	GSE20685	GSE21653
Number	1098	327	252
Age (median)	58	46	55
T	–	–	–
T1	279	101	57
T2	631	188	121
T3	137	26	66
T4	39	12	0
UNKNOW	3	0	8
N	–	–	–
N0	497	137	116
N1	352	87	133
N2	119	63	0
N3	72	40	0
UNKNOW	17	0	3
M	–	–	–
M0	906	319	–
M1	22	8	–
UNKNOW	161	0	–
Stage	–	–	–
Stage I	181	–	–
Stage II	619	–	–
Stage III	246	–	–
Stage IV	20	–	–
UNKNOW	23	–	–
Overall survival Days (median)	1232	1862	1800

We selected shared DEGs between the GEO and TCGA datasets, and the GEO dataset was batch corrected. Differentially expressed genes were selected for prognostic relevance based on univariate Cox regression analysis. OS was defined as death due to BC. This process selected and narrowed down variables by running the *R package “glmnet”* in the regression panel. The survival status of patients in the cohort from TCGA was the regression response variable, and the matrix of intersecting genomes was the independent variable. lasso regression analysis and lasso regression models are used in the analytical models. This analysis is used to select the best number of models and the coefficients of the corresponding genes. Penalty parameters of the panel were calculated using cross-validation, and optimal patient risk scores were calculated by multiplying the gene expression value by its coefficient as follows:

Risk scores=coefficients(A) plus expressed values of gene(A)+coefficients(B)plus expressed values of gene(B)+...+

A prognostic model for genes associated with necroptosis was developed based on patients with TCGA. With the model developed, high-risk patients and low-risk patients were classified according to the median risk value. After that, a validation model was built using GSE21653and GSE20685. The same prognostic model was applied to score the patients. And the performance of a prognostic nomogram was then assessed using Kaplan–Meier curves and the area under the receiver operating characteristic curve (ROC).

### Identification of Independent Prognostic Parameters for BC

SurvStat and riskscores were performed on high-risk and low-risk patients to distinguish the distribution of the different populations. We use univariate and multifactorial regression analyses to assess the ability of risk scores to act as independent predictors (van der Wal et al., 2019). In 38 genes with T-stage, N-stage, age, gender and riskscore were analyzed to demonstrate the genes with the lowest *p*-values. GESA software was used to validate the highly expressed pathways in HRG and LRG. The main enrichment pathways for each of the high-risk and low-risk samples are also plotted. Values with *p < 0.05* were considered statistically significant.

### Acquisition of DMAS-Pharmacological Target and GO and KEGG Enrichment Analysis of Overlapping Genes in BC

The Swiss Target Prediction database (http://www.swisstargetprediction.ch/) was used to predict the possible drug targets connected to DMAS. Overlapping genes between DEGs and drug targets were selected for target docking. GO and KEGG pathway enrichment analysis to demonstrate the pathways affected after drug action.

### Selection of Core Proteins Associated with Necroptosis in BC

The string database (https:// www.string-db.org/) was used to obtain target interaction network maps in protein-protein interactions of the overlapping gene targets. The parameters were analyzed using *Cytoscape sofe*, and the top five proteins in degree ranking were selected for docking with drugs (Otasek et al., 2019). The molecular structure of DMAS was obtained from the PubChem database (https://pubchem.ncbi.nlm.nih), and the structure of the core protein was determined from the PDB database (https://www.rcsb.org/). After obtaining the core protein, the protein molecules and drug molecules are dehydrated and hydrogenated to complete the steps prior to molecular docking. The Drugs and core targets were docked using *AutoDock Vina software*.

### Analysis of TCGA Data for Immune Microenvironment and Chromosomal Mutations

We downloaded data from TCGA on genetic mutations in breast cancer patients and carried out a comprehensive analysis of these data. High-risk and low-risk patients were classified according to the prognostic model, and these two groups were analyzed for gene mutation status. Differences in mutations between these two groups of patients were obtained. The difference in tumor mutation load between HRG and LRG was analyzed, and this difference was expressed as a K-M curve. The proportion of immune cell composition for each patient is also shown. Differences in estimatescore, stromalscore, and immunescore between the high-risk group and low-risk group were analysed. The *R package “CIBERSORT”* was used to analyse five types of immune cell infiltrating cells with survival differences in breast cancer. The *R package “pRRophetic”* was used to compare the drug sensitivity of patients in the high and low-risk groups. Information on drugs and RNAexpress date was downloaded from *CellMinerCBD (* *https://discover.nci.nih.gov/cellminer/home.do* *)* and the top 16 drugs with the highest correlation were selected.

### Statistical Analysis

All data were statistically analyzed using R software *4.1.1.* Student’s t-tests were used to examine differences in gene expression between tumor and adjacent normal tissue. OS was compared between patients at high and low risk using Kaplan–Meier curves. Independent predictors were identified using univariate and multivariate Cox regression analyses. Differences were considered statistically significant at *p < 0.05*.

## Results

### Identifying DEGs and Corresponding Functional Enrichment

**FIGURE 2:**
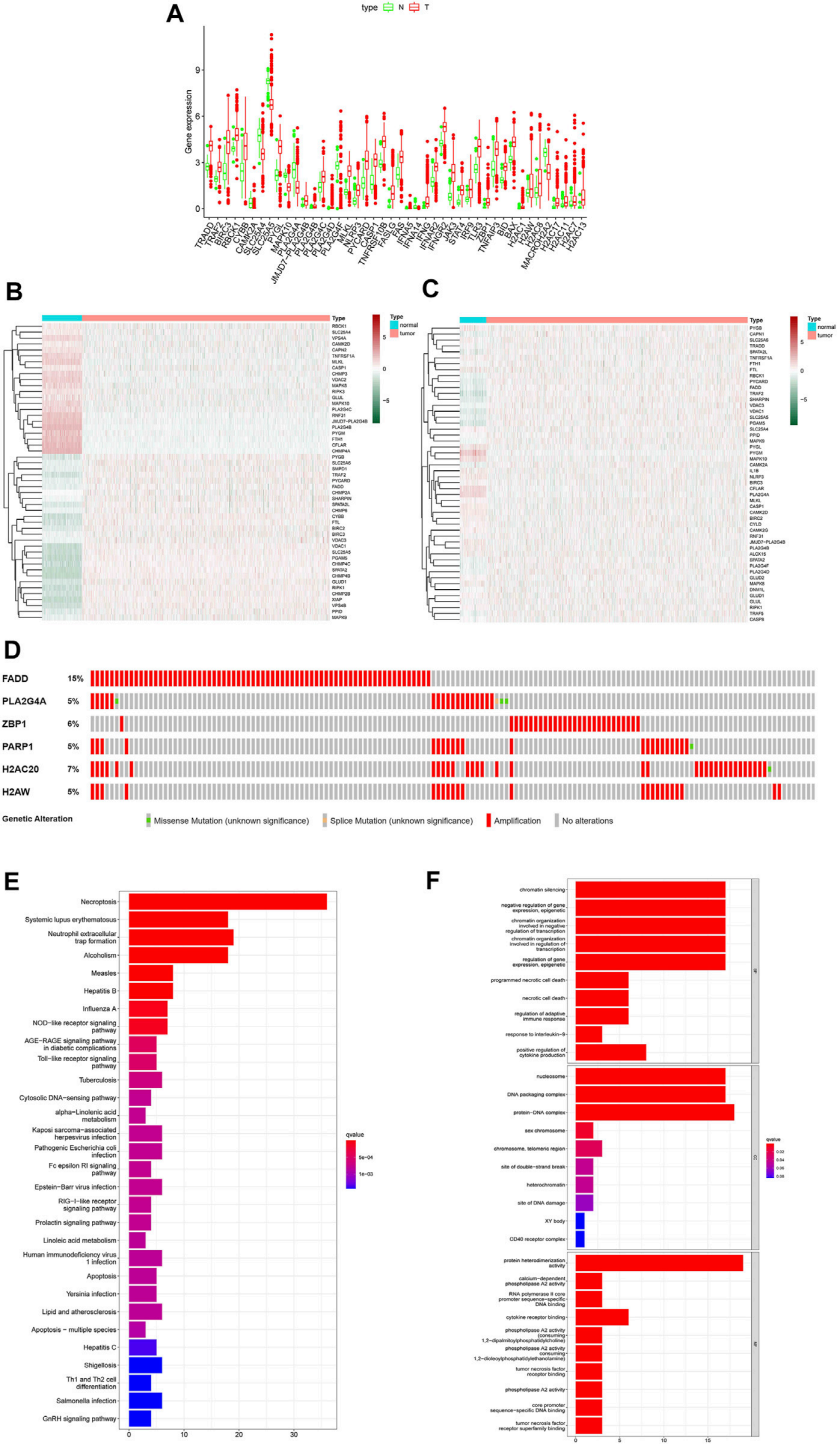
Differential genetic analysis. Box plots indicate genes that are abnormally expressed in tumor tissue compared to adjacent normal tissue **(A)**, Heat map of the top 50 different expressions gene between tumor tissue and GTEx normal breast tissue **(B)**, Heat map of the top 50 different expressions gene between tumor tissue and adjacent normal tissue in patients **(C)**. Probability of chromosomal mutations in differentially expressed genes **(D)**, The top 30 significant terms of KEGG analysis **(E)** The top 30 significant terms of GO function enrichment. BP biological process, CC cellular component, MF molecular function **(F)**.

All necroptosis-related genes that is differented expression in TCGA samples are showed (*FDR < 0.05,* log *FC > 0.5, p < 0.05*, Figure 2A). Heat map shows the differentially expression of the top 50 necroptosis-associated genes in the GTEx and TCGA samples (GTEx N = 179, T = 1109, Figure 2B). The differentially expression of the top 50 necroptosis-associated genes in the TCGA patients was showed (normal = 113,tumor = 1109, Figure 2C). The mutation rates of DEGs with >3% were showed by using cBioPortal (http://www.cbioportal.org/, Figure 2D), and the highest rate of amplification-based mutations was the Fas Associated Via Death Domain (FADD). The genes of expression, including NDGEs, STAT5A, STAT5B, TLR4, PYGL, PYGLM, MAPK10, PLA2G4A, and IL33, are downregulated. The other genes were up-regulated. The mainly KEGG enriched pathways of NDEGs were necroptosis, systemic lupus erythematosus, neutrophil extracellular trap formation, alcoholism, and measles (Figure 2E). Biological process pathways were enriched in chromatin silencing. Cellular component pathways enriched in nucleosomes, DNA packaging complex, protein–DNA complex, and other pathways. The molecular function was enriched in the protein heterodimerization activity pathway (Figure 2F).

### Classification of Individuals

**FIGURE 3:**
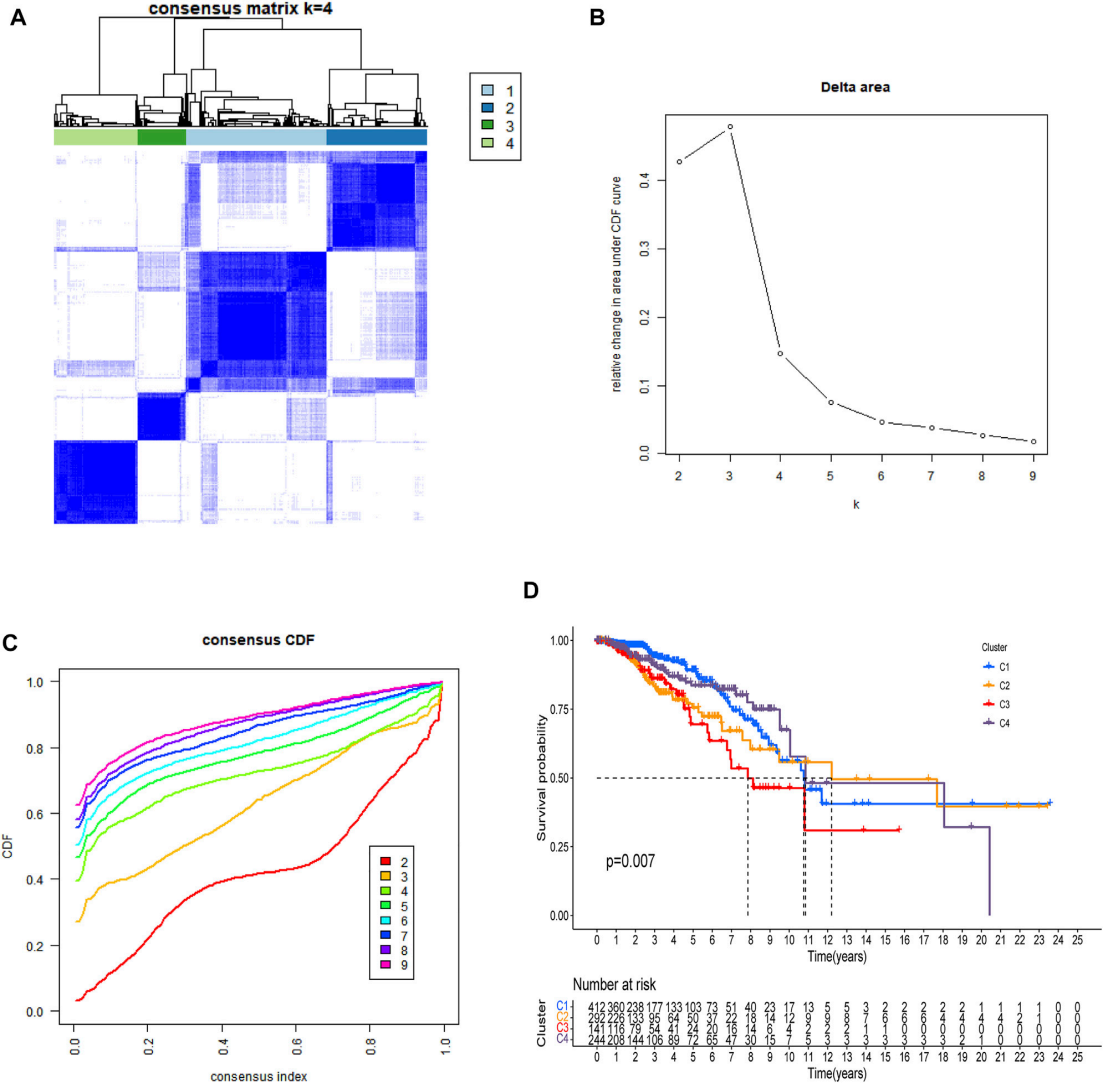
All patients were classified using consensus matrix = 4 **(A)**, delta are <0.2 **(B)**, and consensus CDF = 4 **(C)**, and Kaplan-Meier curve of OS was compared between them **(D)**.

There is a clear difference between these four categories of patients (Figure 3A). There were also showed that a delta area <0.2 (Figure 3B). When n > four, the increase in CDF is not significant (Figure 3C) and Patients with C3 and C2 breast cancer had a worse prognosis than those with C1 and C4 (Figure 3D, *p* = 0.007).

### Development and Validation of the Model with Necroptosis Related Genes

**FIGURE 4:**
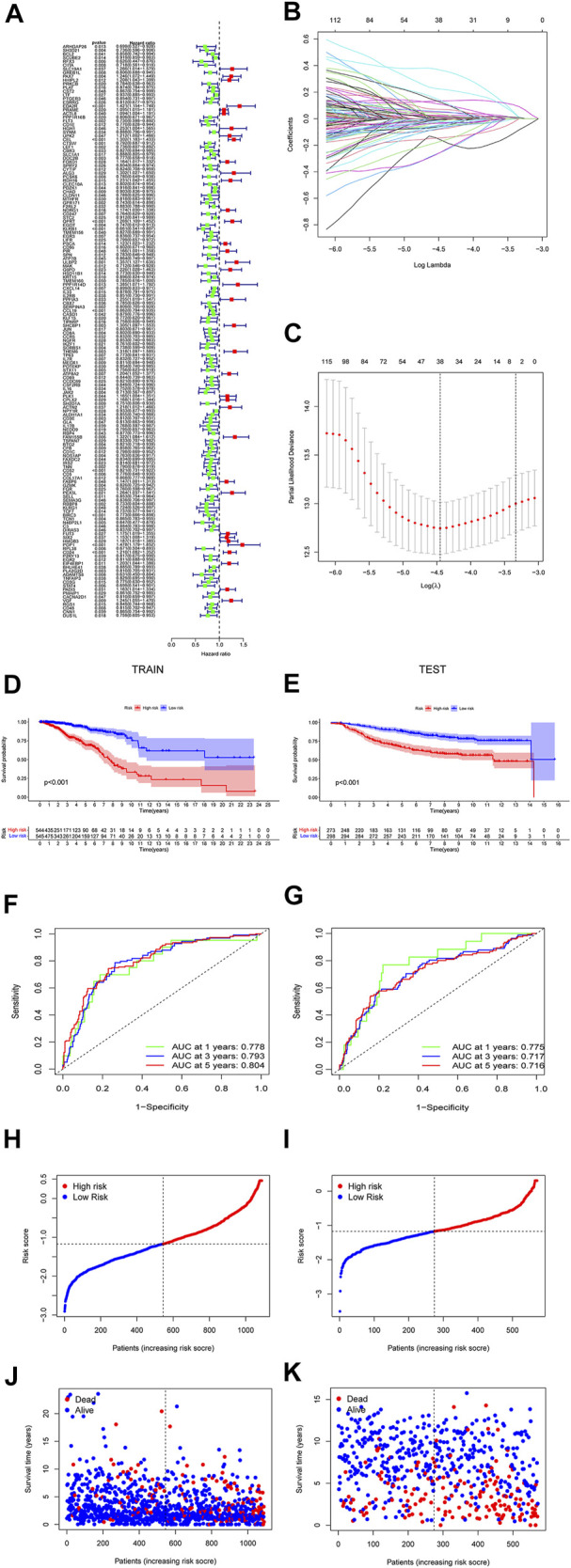
The forest plots indicate genes associated with prognosis-related necroptosis as indicated by the univariate Cox regression analysis **(A)**. Lasso regression analysis and Lasso regression model reduced variable **(B,C)**. Kaplan–Meier curves of OS in the high-risk and the low-risk groups stratified by the necroptosis-related signature in the cohorts **(D,E)**. The number of patients in different risk groups. The ROC analysis of OS for the signature at 1 year, 2 years, and 3 years **(F,G)**. The number of patients in different risk groups. Survival status of patients in different groups **(H,I)**. Survival status of the test patients in different groups **(J,K)**.

The prediction model was made up by the genes with *p* < 0.05 (Figure 4A). Lasso regression (Figure 4B) and lasso cross-validation (Figure 4C) were used to build the prognostic model. Table 2 shows the coefficients of each gene, and the training group shows a significant difference between high and low risk (*p < 0.001,* Figure 4D). The testing group also validated our model with a significant difference (*p < 0.001,* Figure 4E), with higher risk patients having worse OS. The AUCs at 1, 2, and 3 years were 0.778, 0.793, and 0.804 in training group, (Figure 4F). The AUCs at 1, 2, and 3 years reached 0.775, 0.717, and 0.716 in testing group, respectively (Figure 4G). We then demonstrated the riskscore (Figures 4H,I) and survstat (Figures 4J,K) in the training and validation sets and found significant differences between the groups at high and low risk.

**TABLE 2:** All coefficients of the genes in the signature.

Gene	Coef	Gene	Coef
SH3D21	−0.0074455	THEM6	0.020045
RFX3	−0.2490625	POTEKP	−0.00695
PAX7	0.02433956	STX11	−0.05025
PLAT	−0.0774654	ATP8A2	0.107139
ESRRG	−0.071047	NOS1AP	−0.05974
EDA2R	0.36366458	TNN	−0.02845
FLT3	−0.0522195	CD52	−0.00446
CEL	0.13334125	FABP6	0.006832
CBR3	−0.0188455	PEX5L	0.132623
FOXD1	0.01485358	RBBP8	−0.14367
QPRT	0.04831453	TCN1	−0.02457
KLRB1	−0.1649283	SIX2	0.0331
ATP7B	−0.0558802	POP1	0.221766
ULBP2	0.07025883	RPL38	−0.24775
MAK	−0.0116256	CD24	0.008967
KRT15	−0.0343491	EIF4EBP1	0.052484
PPP1R14D	0.11970139	PLA2G2D	−0.05766
SERPINA3	−0.0211194	CACNA2D1	−0.04342
CASD1	−0.0077578	DUS1L	−0.16867

### Independent Prognostic Value of the Risk Score

**FIGURE 5:**
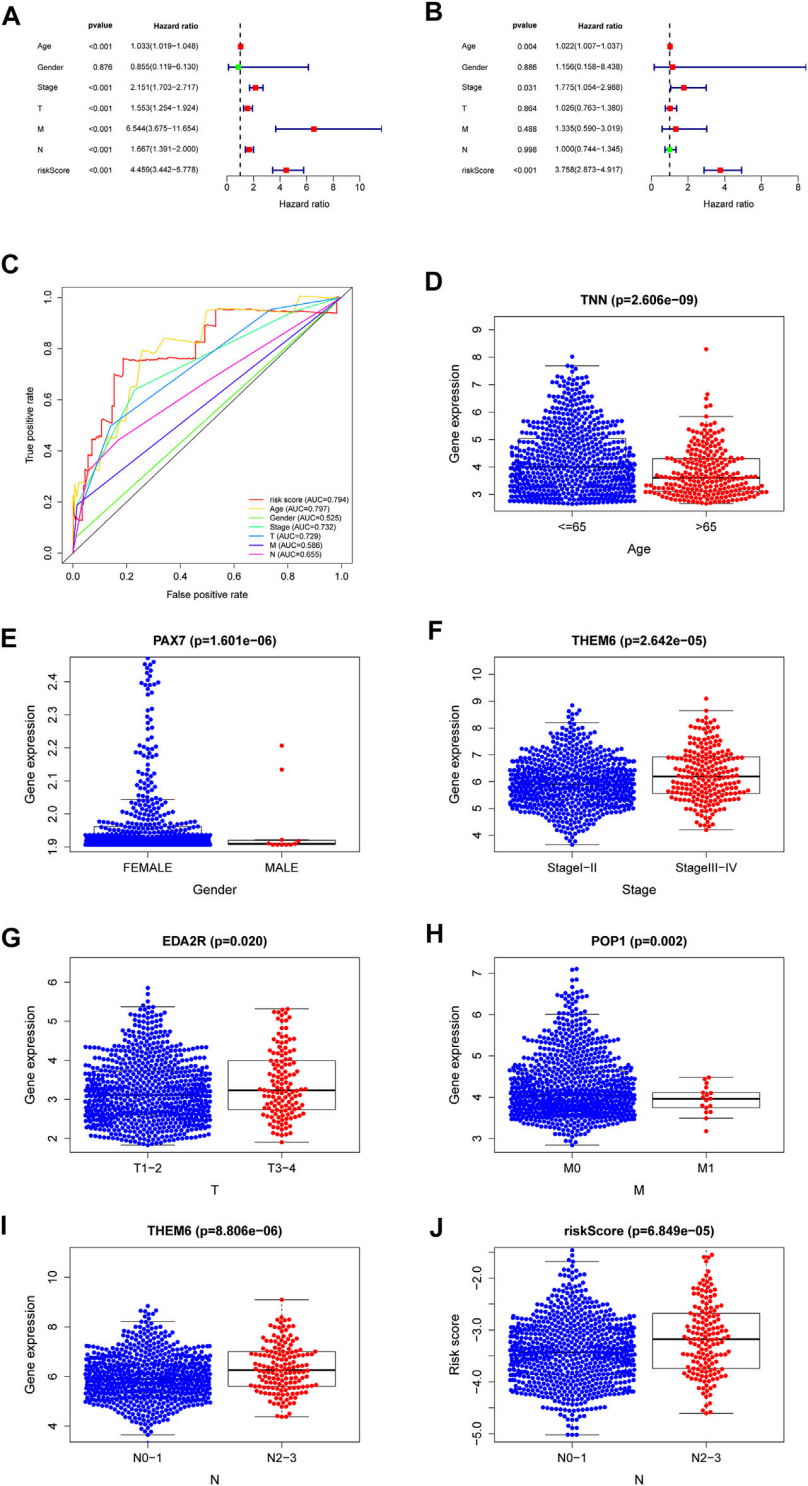
A forest plot of univariate Cox regression analysis in the cohorts **(A)**. A forest plot of multivariate Cox regression analysis in the cohorts **(B)**. The ROC analysis of OS for the signature and the clinicopathologic parameters **(C)**. Necroptosis-related-gene TNN in the cohorts stratified by age **(D)**. Necroptosis-related-gene PAX7 in the cohorts stratified by gender **(E)**. Necroptosis-related-gene THEM6 in the cohorts stratified by stage **(F)**. Necroptosis-related-gene EDA2R in the cohorts stratified by T stage **(G)**. Necroptosis-related-gene POP1 in the cohorts stratified by M stage **(H)**. Necroptosis-related-gene THEM6 in the cohorts stratified by N stage **(I)**. The necroptosis-related signature in the cohorts stratified by N stage **(J)**.

The results of the univariate and multivariate Cox regression analyses of TCGA verified that risk-scores could be used by an independent predictors of OS (*p < 0.001; HR= 4.459; 95% CI:3.442–5.778,* Figure 5A and *p < 0.001, HR = 3.758; 95% CI:2.873–4.917,* Figure 5B, respectively). The results suggest that the riskscore can be used as an independent prognostic factor like other clinical traits to predict the prognosis of patients. The risk-scores were found to be a stronger predictor than others, including age, gender, stage, T-stage and N-stage. The AUC of the risk score was 0.794 (Figure 5C). The risk-scores were higher than the AUC for almost all clinical features (AUC = 0.794), except for lower than age (AUC = 0.797). The reliability of the model was demonstrated. These findings confirmed that the risk score could independently predict the prognosis of patients.

### Relationships Between the NRGs and Clinical

Genes with the lowest *p* values related with individual clinical traits were plotted to verify associations between NRGs and clinical traits. Supplementary Table S1 shows relationships for other genes. The genes with the closest relationships with age, gender, stage, T-stage, N-stage and M-stage were those encoding tenascin N (*TNN*, Figure 5D), paired box protein pax-7 (*PAX7*, Figure 5E), thioesterase superfamily member 6 (THEM6, Figure 5F), ectodysplasin A2 receptor (*EDA2R*, Figure 5G), ribonucleases P/MRP protein subunit (*POP1*, Figure 5H), and *THEM6* (Figure 5I), respectively. The higher the riskscore, the higher the lymph node grade (Figure 5J). Univariate analysis associated risk scores with several clinical traits (Supplementary Table S1).

### Pathway Validationby GSEA and Immune Microenvironment

**FIGURE 6:**
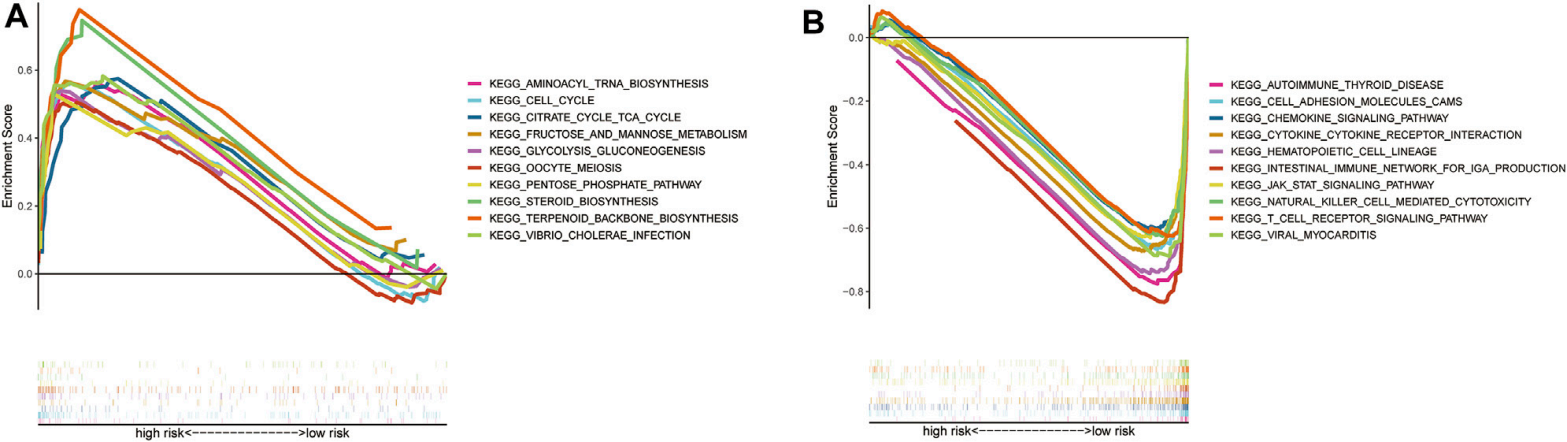
GSEA analysis results **(A)**. GSEA validated the top 10 of enhanced activity in high-risk group **(B)**. GSEA validated the top 10 of the downgrade in low-risk group **(C)**.

The individual pathways of patients at high risk were mainly tumor cell metabolism, survival, and cell cycle-related, such as aminoacyl tRNA biosynthesis, and cell cycle progression (Figure 6A). In contrast, those patients with low risk were mainly downregulated immunological diseases, tumor cell metastasis, promotion of tumor cell proliferation, and other related pathways (Figure 6B).

**FIGURE 7:**
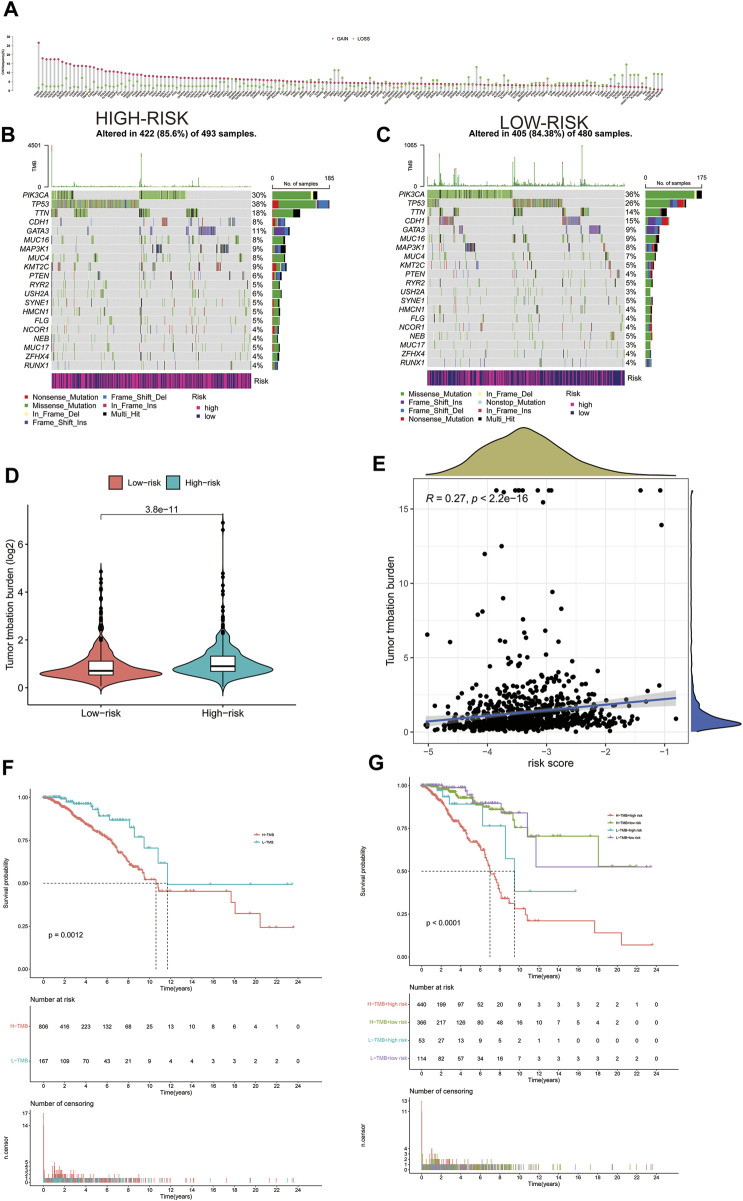
Gain and loss of all differential genes **(A)**. Waterfall plot of each mutation in the high-risk group in TCGA **(B)**. Waterfall plot of each mutation in the low-risk group in TCGA **(C)**. The difference of Tumor Mutation Burden between high-risk and low-risk groups **(D)**. Correlation between tumor mutation load and risk score **(E)**. Kaplan–Meier curves of OS between high and low tumor mutation load **(F)**. Kaplan–Meier curves of OS between high and low tumor mutation load and high- and low-risk groups **(G)**.

Gain and loss analysis of NDEGs showed that *FADD* had the highest frequency of copy number variations, and all genes tended to have a copy number variation gain (Figure 7A). We then analyzed different gene mutations between the patients at high and low risk and found that *tumor protein P53 (TP53)* was the highest mutation in those at high-risk groups (Figure 7B), whereas it was *phosphatidylinositol-4,5-bisphosphate 3-kinase catalytic subunit alpha (PI3KCA)* at low-risk groups (Figure 7C). A comparison of the two groups then revealed a higher tumor burden in the HRG (Figure 7D). We then categorized the patients according to whether they had a high (H-TMB) or a low (L-TMB) tumor burden and found it that was significantly different OS rates (*p = 0.0012*, Figure 7F). We further categorized the patients based on risk scores and tumor load to evaluate survival differences and found that those at high risk with a high tumor burden had the worst OS (*p < 0.0001*, Figure 7G). Tumor load positively correlated with risk score (Figure 7E *, p < 2.2e-16*, *R = 0.27*), validating that tumor load was indeed associated with our risk model (Figure 7E). Supplementary Figures S1–S5 demonstrate the immune microenvironment and screening of chemotherapeutic agents in breast cancer.

### Screening out Core Targets Docking to DMAS

**FIGURE 8:**
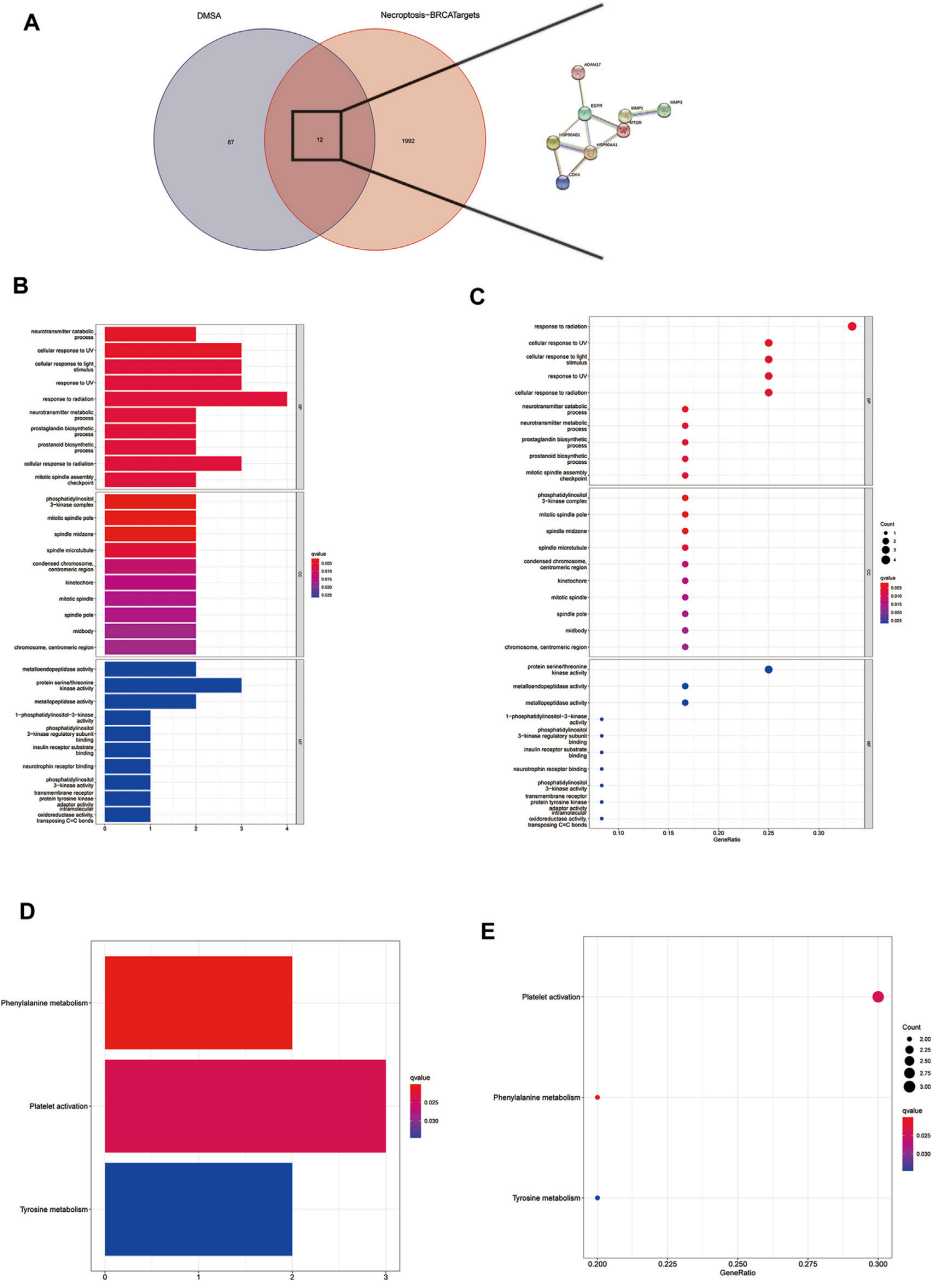
Screening for intersecting genes of drug targets and differentially expressed genes **(A)**. The top 30 significant terms of GO function enrichment. BP biological process, CC cellular component, MF molecular function **(B,C)**. The all significant terms of KEGG analysis **(E,F)**.

**FIGURE 9:**
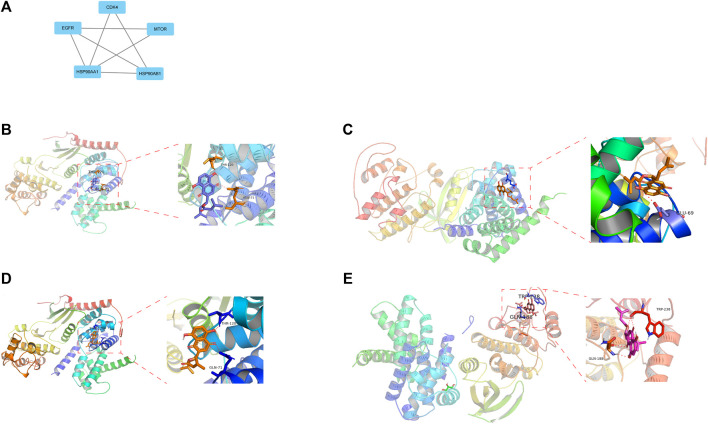
Five core drug targets **(A)**, DMSA docked with the 6p8g **(B)**, 2w9z **(C)**, 6p8e **(D)**, and 2w96 **(E)** conformations of CDK4D.

There are a total of 12 overlapping genes between the docking targets of DMAS and DEGs (Figure 8A), and the GO and KEGG pathway enrichment of overlapping genes was then analyzed. The GO pathways were mainly enriched in photoresponse, phosphatidylinositol 3-kinase complex, cytokinesis, and metalloendopeptidase activity (Figures 8B,C). The KEGG pathway was enriched in the metabolism of key amino acids (Figures 8D,E). We used Cytoscape to select five core targets based on degree, including *epithelial growth factor receptor (EGFR)*, heat shock protein 90 alpha family class B member 1 (*HSP90AA1*), 90 alpha family class B member 1 (*HSP90AB1*), mTRO, and cyclin-dependent kinase 4 (*CDK4*) (Figure 13A). We docked drugs to core targets using AutoDock. We found that *CDK4* had the highest connecting energy and docked all four of its conformations as follows: 6p8e and 6p8g docked to amino acid residues Thr-120 and Gln-71 via hydrogen bonds (Figures 9B,D), 2w9z docked to Glu-69 (Figure 9C), 2w96 docked to Trp-238 and Gln-188 connected to a hydrogen bond (Figure 9E). Thus, we concluded that DMAS could stably dock with *CDK4* via Thr-120 and Gln-71.

## Discussion

BC accounts for about 30% of all cancers in women, with a 15% ratio of mortality to incidence (Siegel et al., 2020). Over 40,000 women died of BC, while >270,000 patients were diagnosed for the first time with BC in the United States in 2018 (Siegel et al., 2019). The prognosis of different types of breast cancer is different. Breast cancers of the same molecular staging can also lead to differences in OS between patients due to tumor heterogeneity. A high degree of BC heterogeneity implies that patients with similar clinical features might have very different prognoses. Therefore, other factors must be considered and integrated to guide clinical treatment and enhance the prognosis of patients with BC. Necroptosis, a caspase-independent form of regulatory cellular apoptosis, is caused by genotoxic stress and activation by various anticancer drugs and thus offers a new strategy for treating drug-resistant cancer cells (Della Torre et al., 2021). It is mainly metabolized in cells *via* Toll-like receptors (He et al., 2011), T cells (Ch’en et al., 2011), interferon, and the TNF receptor superfamily (Lu et al., 2014). To distinguish between necrosis caused by physical trauma, regulatory necrosis is referred to as programmed necrosis or necroptosis (Moriwaki and Chan, 2013). As far as we can ascertain, the prognosis of patients with BC has never been predicted based on necroptosis.

We developed a new NRG signature for predicting OS in patients with BC by analyzing the TCGA and GEO databases. Patients at low risk survived longer than those at high risk. The reliability of our findings was confirmed using ROC curves, PCA and t-SNE analyses, and an independent GEO dataset. We found higher immune functions and immune checkpoints, as well as more immune enrichment pathways in patients at low risk than in those at high risk. These findings mean that an important cause of the low OS among patients at high risk is the nature of immune factors. Immune cells mainly comprise CD8^+^ T, CD4^+^ T, regulatory T, myeloid suppressor, and NK cells, as well as tumor-associated macrophages and neutrophils, all of which interact to exert anti- or pro-tumor effects. In contrast, increased expression of pro-tumor cells, such as regulatory T cells, tumor-associated macrophages, tumor-associated neutrophils, and myeloid-derived suppressor cells usually predicts a poorer prognosis.

Among our signatures, *SH3 domain-containing protein 21 (SH3D21)* might serve as a target to improve the effects of gemcitabine in the treatment of pancreatic cancer (Masoudi et al., 2019). *Regulatory factor X3 (RFX3)* is associated with predisposition genes in endometrial cancer (Shivakumar et al., 2019), metastasis in BC(Legare et al., 2017), chromosomal rearrangement in B lymphocytes (Twa et al., 2015), and the promotion of cancer cell proliferation (Ham et al., 2019). Ectodysplasin A2 receptor is associated with prognosis in BC patients (Deryusheva et al., 2017). *Adenosine triphosphatase phospholipid transporting 8A2 (ATP8A2)* participates in gene methylation, reduces mRNA expression, and alters expression to disrupt the differentiation state of precancerous cells in various cancers, including BC (Ohara et al., 2017). *Peroxisomal biogenesis factor 5 like (PEX5L)* can be an independent prognostic factor for gastric cancer progression (Fang et al., 2021). Ribonucleases P/MRP protein subunit can act as an oncogene in maintaining cell viability and accelerating BC cell metastasis (Liu et al., 2021), providing a regulatory feedback loop that shuts down excessive inflammatory responses, thus blocking systemic inflammatory responses (de Almeida et al., 2015). *Ribosomal protein L38 (RPL38)* controls proliferation and apoptosis in gastric cancer through the miR-374b-5p/vascular endothelial growth factor (*VEGF*) signaling pathway (Ji and Zhang, 2020) and is overexpressed in a resistant BC ductal cell line (Hurvitz et al., 2015). The cluster of differentiation CD8^+^ T cells in early recurrent hepatocellular carcinoma is characterized by killer cell lectin-like receptor B1 (*KLRB1; CD161*) overexpression, mainly in an innate class, low cytotoxicity, low clonal expansion state, and low expression of costimulatory and checkpoint molecules (Sun et al., 2021). We assessed an NRG signature for BC treatment and established an NRG signature and nomogram of OS that could reliably and reproducibly determine the prognosis of patients with BC.

The immune microenvironment is an important factor in BC. A very low-energy diet decelerates tumor progression, whereas a high-fat diet promotes the progression of malignant tumors (Sun et al., 2020). The metabolism of tumor cells can influence the immune microenvironment, ultimately affecting tumor progression. Tumor cells compete with immune cells for glucose, produce more lactic acid, form an acidic tumor microenvironment, inhibit immune cell functions, and lead to the occurrence and development of tumors. The previous findings showed that the metabolism of immune cells changes when stimulated to differentiate into various functional types, which is similar to tumor cell metabolism. The metabolism of tumor cells causes changes in the tumor microenvironment, resulting in a decrease in immune cell function and the formation of cancer. We found that the types of infiltrative immune cells significantly differed between the patients at high and low risk; immune functional status is better in the LRG. The immune microenvironment might contribute to the inconsistent OS between these two groups of patients. We plan to investigate how metabolic changes in tumor cells and immune cells affect the progression of BC. We found that the treatment component was missing from all prognostic models now. But improving the patient’s OS through treatment is our aim, hence the association to link the two components through DEGs.

In our study, a prognostic model of necroptosis genes was developed to predict the prognosis of breast cancer patients, and drugs were used to treat the genes associated with necroptosis. However, our study did not validate our analysis through a complete experimental process and the underlying DMAS trial for breast cancer was not demonstrated. In our group, however, we have demonstrated a clear therapeutic effect of DMAS in triple-negative breast cancer, and the mechanism of DMAS treatment in breast cancer will be shown in full in a separate article. This is the biggest gap in this study and an area for improvement.

## Conclusion

This study builds a prognostic model through the differential expression of genes in necroptosis, a model that efficiently predicts survival time of patients, while applying DMAS to target targets in breast cancer. By establishing a new research paradigm: the combination of prognostic modelling and network pharmacology.

## Data Availability

The datasets presented in this study can be found in online repositories. The names of the repository/repositories and accession number(s) can be found in the article/Supplementary Material.
