# Supplementary figures and images for: A Novel Approach: Combining Prognostic Models and Network Pharmacology to Target Breast Cancer Necroptosis-Associated Genes
Source: Front Genet. 2022 Aug 22;13:897538. doi: 10.3389/fgene.2022.897538 (PMC9441943; doi:10.3389/fgene.2022.897538)

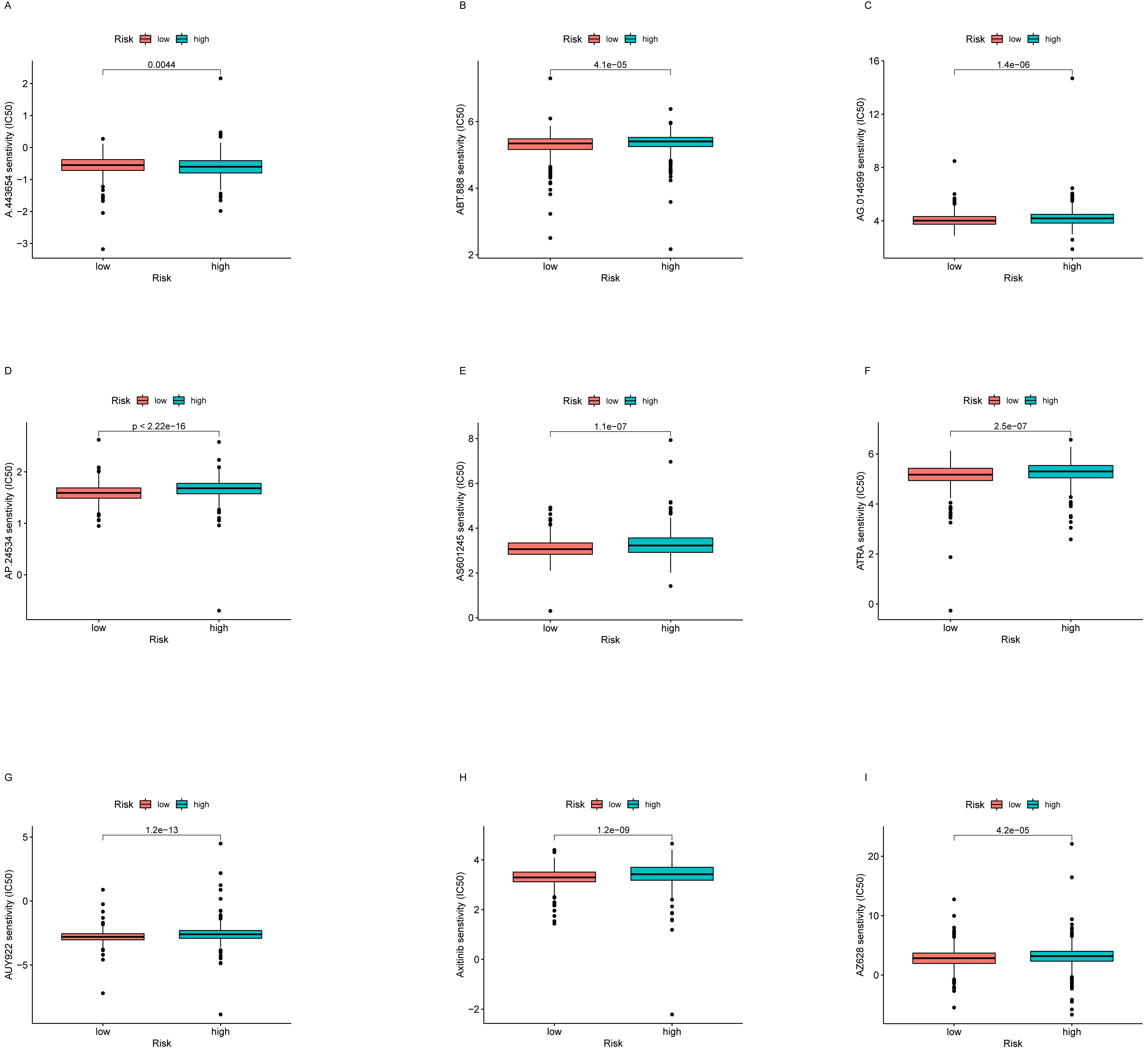

Supplement: Supplementary file 2 [file Image4.TIF]

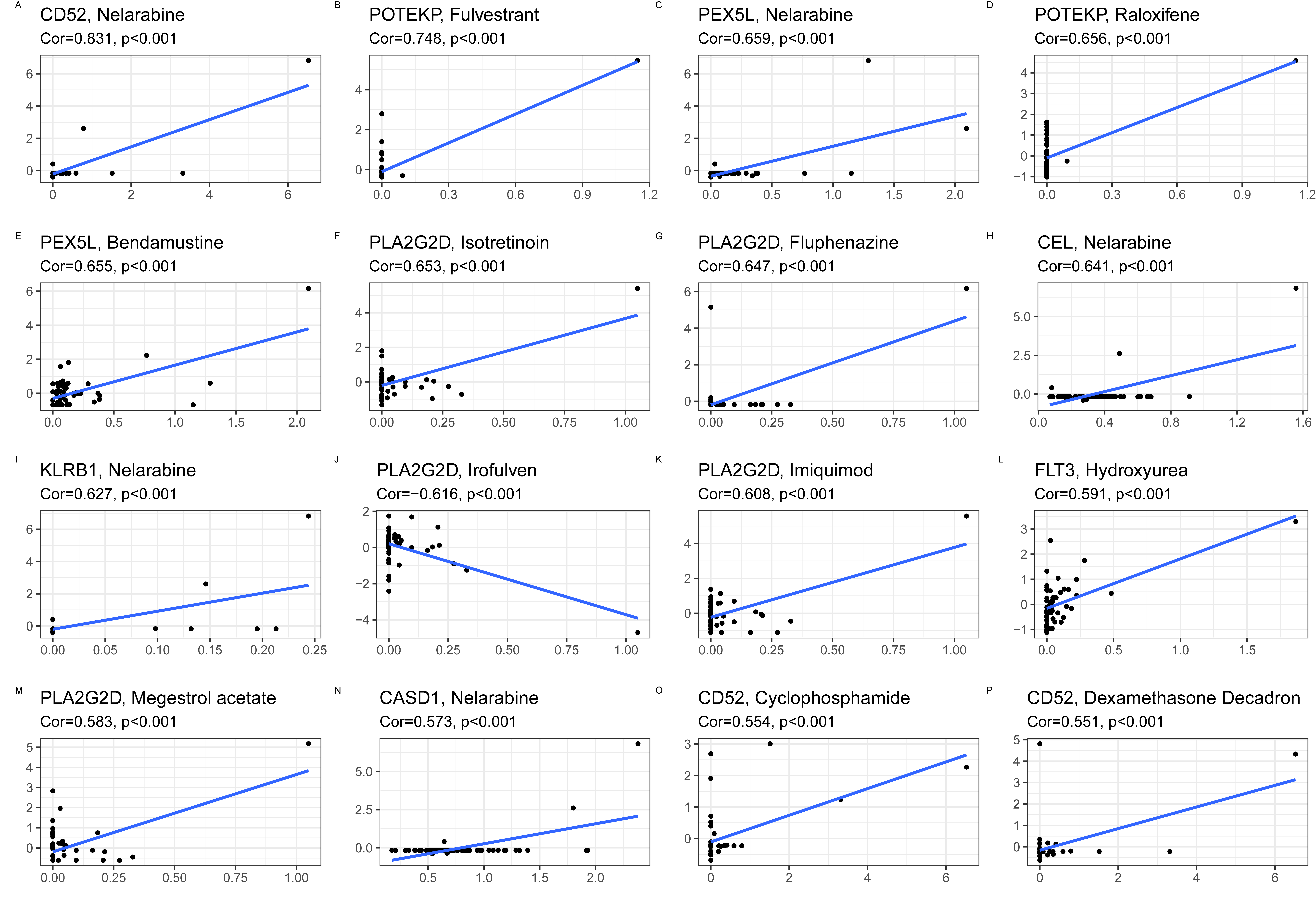

Supplement: Supplementary file 5 [file Image5.TIF]
