# Supplementary material for: A Novel Approach: Combining Prognostic Models and Network Pharmacology to Target Breast Cancer Necroptosis-Associated Genes
Source: Front Genet. 2022 Aug 22;13:897538. doi: 10.3389/fgene.2022.897538 (PMC9441943; doi:10.3389/fgene.2022.897538)
Supplement: Supplementary file 6 [file DataSheet1.docx]

Supplementary Material

# Supplementary Table 1

Supplementary Table 1

| Gene | Age | Gender | Stage | T | M | N |
| --- | --- | --- | --- | --- | --- | --- |
| RFX3 | 0.023 | 0.184 | <0.001 | 0.187 | 0.491 | 0.003 |
| PAX7 | 0.421 | <0.001 | 0.303 | 0.853 | 0.939 | 0.107 |
| ESRRG | 0.182 | 0.889 | 0.361 | 0.948 | 0.147 | 0.586 |
| EDA2R | 0.013 | 0.189 | 0.006 | 0.020 | 0.873 | 0.015 |
| FLT3 | 0.188 | 0.400 | 0.010 | 0.037 | 0.173 | 0.006 |
| CEL | 0.894 | <0.001 | 0.325 | 0.406 | 0.701 | 0.199 |
| CBR3 | 0.002 | 0.029 | 0.231 | 0.302 | 0.805 | 0.900 |
| KLRB1 | <0.001 | 0.014 | 0.503 | 0.917 | 0.242 | 0.399 |
| ULBP2 | 0.256 | <0.001 | 0.566 | 0.229 | 0.870 | 0.995 |
| MAK | 0.555 | 0.004 | 0.219 | 0.536 | 0.062 | 0.429 |
| KRT15 | 0.038 | 0.498 | 0.111 | 0.924 | 0.041 | 0.161 |
| PPP1R14D | 0.095 | 0.702 | 0.041 | 0.750 | 0.493 | 0.106 |
| THEM6 | 0.327 | 0.887 | <0.001 | 0.187 | 0.992 | <0.001 |
| POTEKP | 0.328 | 0.020 | 0.906 | 0.286 | 0.074 | 0.627 |
| TNN | <0.001 | 0.048 | 0.091 | 0.111 | 0.185 | 0.120 |
| CD52 | 0.004 | 0.186 | 0.767 | 0.158 | 0.328 | 0.176 |
| FABP6 | 0.099 | 0.146 | 0.512 | 0.508 | 0.275 | 0.483 |
| PEX5L | 0.007 | 0.823 | 0.242 | 0.993 | 0.453 | 0.128 |
| RBBP8 | 0.520 | 0.231 | <0.001 | 0.293 | 0.038 | <0.001 |
| TCN1 | 0.015 | 0.737 | 0.018 | 0.125 | 0.095 | 0.055 |
| POP1 | 0.137 | 0.519 | 0.231 | 0.037 | 0.002 | 0.915 |
| RPL38 | 0.057 | 0.033 | 0.961 | 0.886 | 0.748 | 0.274 |
| PLA2G2D | 0.012 | <0.001 | 0.433 | 0.286 | 0.328 | 0.728 |
| CACNA2D1 | <0.001 | 0.013 | 0.670 | 0.853 | 0.873 | 0.548 |
| DUS1L | 0.019 | 0.005 | 0.951 | 0.493 | 0.729 | 0.357 |
| RiskScore | <0.001 | 0.325 | <0.001 | 0.217 | 0.042 | <0.001 |
